# Functional Preservation and Oncologic Control following Robot-Assisted versus Laparoscopic Radical Prostatectomy for Intermediate- and High-Risk Localized Prostate Cancer: A Propensity Score Matched Analysis

**DOI:** 10.1155/2021/4375722

**Published:** 2021-12-21

**Authors:** Wen Deng, Ru Chen, Ke Zhu, Xiaofeng Cheng, Yunqiang Xiong, Weipeng Liu, Cheng Zhang, Yulei Li, Hao Jiang, Xiaochen Zhou, Ting Sun, Luyao Chen, Xiaoqiang Liu, Gongxian Wang, Bin Fu

**Affiliations:** ^1^Department of Urology, The First Affiliated Hospital of Nanchang University, Yongwai Street 17, Nanchang, Jiangxi Province, China; ^2^Jiangxi Institute of Urology, Yongwai Street 17, Nanchang, Jiangxi Province, China; ^3^Department of Urology, The First Hospital of Putian City, Putian, Fujian Province, China

## Abstract

**Aim:**

To evaluate the urinary continence (UC), erectile function, and cancer control obtained following robot-assisted radical prostatectomy (RARP) and laparoscopic radical prostatectomy (LRP) for intermediate- and high-risk localized prostate cancer (PCa).

**Materials and Methods:**

232 patients bearing intermediate- and high-risk localized PCa were enrolled in this study. Perioperative, functional, and oncological outcomes were analyzed after applying the propensity score matched method.

**Results:**

Within the matched cohort, the RARP group was corrected with a significantly shorter mean operative time than the LRP group (*p* < 0.001). Patients in the RARP arm were also at a lower risk of ≤ Grade II complications than those in the LRP group (*p* = 0.036). Meanwhile, the proportions of transfusion and ≥ Grade II complications in the RARP group were similar to those in the LRP group (*p* = 0.192 and *p* = 1.000, respectively). No significant differences regarding the rates of pT3 disease and positive surgical margin existed between the two groups. RARP versus LRP tended to a significantly higher percentage of UC recovery within the follow-up period. Significant differences were also found between the RARP and LRP arms in terms of erectile function at postoperative 6 months and the last follow-up (*p* = 0.013 and *p* = 0.009, respectively). Statistical comparability in biochemical recurrence-free survival was observed between the two groups (*p* = 0.228).

**Conclusions:**

For the surgical management of intermediate- and high-risk localized PCa, RARP tended to a lower risk of ≤ Grade II complications and superior functional preservation without cancer control being compromised than LRP.

## 1. Introduction

Prostate cancer (PCa), the second most common cancer and the fifth dominating cause of cancer-specific mortality among men around the world [1, 2], has been increasingly discovered due to the widespread diffusion of prostate-specific antigen (PSA) screening, markedly the localized ones [3–5]. Given the slowly evolving nature of localized prostate tumors, PCa destined to cause clinical symptoms or metastases must be distinguished from the more clinically indolent PCa, which is highly unlikely to affect survival and reduces the overtreatment in patients with PCa without compromising opportunities for cure. The D'Amico risk classifications, which were proposed on the basis of clinical and pathological characteristics, including clinical stage, PSA, and biopsy Gleason score, have been widely endorsed [4]. The role of radical prostatectomy (RP) in managing D'Amico low-risk PCa raises concern due to the similar survival benefits for patients with low-risk localized PCa following intermediate radical treatment and active surveillance and RP-related harms to the quality of life [4, 6, 7]. Meanwhile, RP shows a tendency towards an improved survival benefit over watchful waiting for intermediate-risk localized PCa [6, 8, 9]. The 10-year PCa-specific survival rates after RP coupling with node dissection were generally over 90% and remarkably consistent across all large studies focusing on high-risk PCa [10]. In 2020, surgery was not recommended in patients with low-grade, low-volume, or Gleason 6 PCa, in consideration of the minimal clinical benefit and substantial adverse effects following surgery, while RP was considered as an appropriate choice for men with intermediate- and high-risk disease [11].

With the superiority of surgical robots in a three-dimensional magnified vision of the surgical field, improved dexterity, and high precision during the surgical procedure, robot-assisted RP (RARP) is considered a great evolution of minimally invasive surgery to reduce the difficulty associated with complex laparoscopic surgery [12], and it has been widely disseminated for localized PCa since 2001 [13]. However, given the prohibitively high cost of robotic systems and the scarcity of scientific evidence supporting RARP over laparoscopic RP (LRP), the latter was still routinely performed for localized PCa at many centers in Europe and Asia [14, 15]. Furthermore, thus far, whether the advantage of RARP over LRP mentioned above could translate into superior functional preservation and oncological control remains inconclusive due to the scarcity of high-level evidence comparing RARP and LRP for localized PCa [12, 16]. Only three randomized controlled trials concentrated on comparing RARP and LRP for localized PCa, with different endpoints over short-term study periods [17–19], which is far from reaching a convincing consensus about the controversy. In addition, no study focusing on the comparison between RARP and LRP for patients with intermediate- and high-risk localized PCa has been reported yet, while cogent evidence comparing the efficacy and safety of RARP and LRP for intermediate- and high-risk localized PCa is of great clinical importance.

To close this gap of cogent evidence concerning the functional and oncological efficacy of RARP and LRP for intermediate- and high-risk localized PCa, we designed this first analysis comparing RARP and LRP for intermediate- and high-risk localized PCa to document differences in the perioperative, functional, and oncological outcomes obtained after applying the two techniques.

## 2. Materials and Methods

A preprint of an earlier version of our study has previously been published in Research Square [20]. With the approval of the Institutional Review Board and the Ethics Committee of the First Affiliated Hospital of Nanchang University, the prospectively maintained database was meticulously reviewed to retrospectively gather all the demographic, clinical, and pathologic information of patients harboring localized PCa between January 2016 and October 2019. All patients with PCa were screened and incorporated into the final analysis on the grounds of the following eligibility criteria: (1) total serum PSA ≥10 ng/mL or Gleason score ≥7 or localized T2b or T2c stage; (2) patients undergoing RARP or LRP for localized PCa; (3) no clinical evidence of positive lymph nodes or clinical T3-4 stage. Meanwhile, patients with D'Amico low-risk PCa, contraindications for RP, neoadjuvant hormone therapy, or suspected extracapsular extension in the preoperative evaluation were excluded from this study. Only when the case simultaneously satisfied all these criteria were the men included. On the basis of the criteria described above, included in the final analysis were 232 patients, of whom 137 and 95 were divided by surgical approaches into the RARP and LRP arms, respectively. Prostate magnetic resonance imaging and bone scintigraphy were routinely performed in all patients before surgeries.

All surgeries were conducted via the anterior approach by three highly experienced hands (Fu B, Wang GX, and Sun T). Prior to the study initiation, each of these three surgeons had performed more than 300 LRPs and 100 RARPs as an operator or a trainee. The patient assignment was usually at the discretion of these three highly experienced surgeons in accordance with tumor and patient characteristics. Written informed consent was acquired from each patient in both groups. The anterior approach to RARP was done on a multiport basis with the da Vinci Si system in compliance with the techniques established by Menon et al. [21], while the anterior approach to LRP was completed following the surgical steps described by Touijer et al. [22]. All instances in both groups routinely underwent RP with posterior reconstruction. Extended pelvic lymph node dissection (PLND) was routinely performed in all high-risk patients and in those intermediate-risk cases with a preoperative estimated risk exceeding 5% in nodal involvement, while the nodal dissection could be omitted at a low risk of missing positive nodes in other intermediate-risk men. A standardized extended PLND template, including removal of the nodes overlying the external iliac artery and vein, the nodes within the obturator fossa, the nodes medial and lateral to the internal iliac artery, and the nodes overlying the common iliac artery and vein up to the ureteral crossing, was utilized in all cases receiving lymph node dissections. Nerve sparing was preoperatively proposed in consideration of clinical features and intraoperatively modified based on evidence of bundle invasion. Adjuvant radiotherapy was delivered following surgery in patients harboring aggressive disease characteristics (i.e., pT3-4 stage, positive surgical margin (PSM), or lymph node invasion) at final pathology.

Preoperative variables included age, body mass index (BMI), diabetes mellitus, hypertension, American Society of Anesthesiologists (ASA) score, preoperative total PSA, preoperative erectile function quantified in accordance with the International Index of Erectile Function-5 (IIEF-5) score [23], risk stratification assessed with D'Amico risk classifications [4], clinical TNM stage, biopsy Gleason score, and prostate volume calculated by transrectal ultrasound. Perioperative outcomes included operative time (OT), estimated blood loss (EBL), ePLND, nerve sparing procedure, open conversion, transfusion, postoperative hospital stay, postoperative complications evaluated with Clavien–Dindo classification [24], and pathologic results.

Postoperative follow-up was regularly arranged every 3 months within the first year after surgery and every 6 months from the second year after surgery for each patient. Each case routinely underwent postoperative PSA tests every 3 months to monitor the biochemical recurrence (BCR), which was considered as the occurrence that two consecutive rising serum PSA values measured on two separate occasions were 0.2 ng/mL or greater after prostatectomy [2, 25]. BCR-free survival was regarded as the interval length from the date of surgery to that of BCR. Urinary continence (UC) was considered as no use of pads or use of a single safety pad within 24 hours [2, 25]. A safety pad was defined as “no involuntary loss of urine, but a pad was still used.” In the meantime, urinary incontinence was defined as the use of > one pad within 24 hours. The interval length from the date of catheter removal to that of restoration of continence was evaluated by assessing the pads used daily by each patient. The IIEF-5 score questionnaire was routinely completed by each patient receiving RP before surgery and at each postoperative follow-up visit. Full erectile function recovery was defined as an IIEF-5 score ≥17 [26]. The total PSA level and erectile function score were reported at postoperative 6 months and the last follow-up, while the rate of UC recovery was presented at the removal of the catheter, postoperative 6 months, and the last follow-up.

The propensity score matching (PM) method was employed to impose restrictions on significant differences in preoperative clinical and tumor characteristics. The propensity score was calculated with nonparsimonious multivariate logistic regression, with treatment assignment used as a dependent variable and all preoperative variables forced as covariates, namely, age, BMI, diabetes mellitus, hypertension, ASA score, preoperative total PSA, preoperative IIEF-5 score, risk classification, clinical TNM stage, biopsy Gleason score, and prostate volume. The patients in the RARP group were matched to those in the RLRN group at a 1 : 1 ratio by using the nearest neighbour pairing method within the matching strategy.

All continuous variables were analyzed using the Wilcoxon rank-sum test or independent *t*-test. All categorical variables were calculated with Pearson chi-squared test or Fisher's exact test. The Kaplan–Meier method was employed to estimate BCR-free survival probabilities and the proportions of postoperative UC recovery with a log-rank test. All statistical analyses were conducted on STATA version 12.0 (STATA corp., College Station, TX), and statistical significance was defined as a two-sided *p* value of <0.05.

## 3. Results

All preoperative information concerning the clinical and tumor characteristics before and after PM were described in detail (Table 1). Exactly 232 patients with primary intermediate- and high-risk localized PCa in line with the inclusion criteria, comprising 137 RARPs and 95 LRPs, were enrolled in this analysis over the study period being reviewed. All the significant differences in preoperative variables disappeared within the well-balanced matched cohorts after the application of the PM method (Table 1). Finally, except for 10 patients missing an appropriate pair, all the remaining cases in the LRP arm were successfully matched to 85 patients in the RARP group.

Table 2 delineates the perioperative and pathological outcomes in detail. Within the matched settings, no surgery was converted to an open approach in either arm. The patients in the RARP group had a significantly shorter mean OT than those in the LRP group (146.0 versus 167.9 min, *p* < 0.001). However, no significant difference was found in the mean EBL between the two groups (152.6 versus 166.4 mL, *p* = 0.200). A total of 55 (64.7%) and 50 (58.8%) cases underwent ePLND in the RARP and LRP groups, respectively (*p* = 0.430). Meanwhile, the nerve sparing technique was more frequently completed in patients undergoing RARP than those who underwent LRP (48.2% versus 32.9%, *p* = 0.042). No significant differences were observed in the probability of transfusion and > Grade II postoperative complications between the two groups (*p* = 0.192 and *p* = 1.000, respectively). The distributions of pathologic T2 and T3 disease were comparable between the RARP and LRP groups (*p* = 0.345), and the comparability between the two groups remained with regard to the median specimen Gleason score and hospital stay length (*p* = 0.179 and *p* = 0.563, respectively). The median number of lymph nodes removed in the RARP and LRP groups was 10 and 9, respectively (*p* = 0.523). The occurrence rates of PSM and positive lymph node were also statistically similar between the RARP and LRP arms (*p* = 0.260 and *p* = 0.501, respectively). However, the patients in the LRP group had a tendency towards a higher incidence of ≤ Grade II complications than those in the RARP group (*p* = 0.036). 37 (43.5%) and 32 (37.6%) patients received adjuvant radiotherapy in the RARP and LRP groups, respectively (*p* = 0.435).

**Figure 1:**
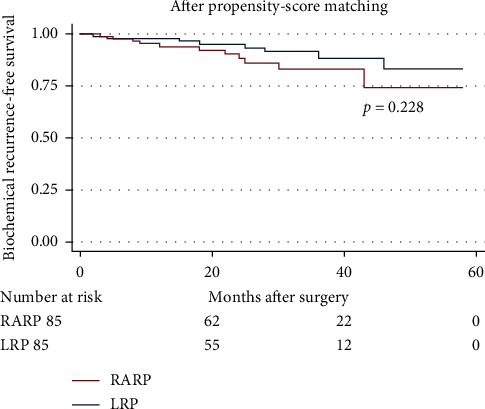
Kaplan–Meier curves showing biochemical recurrence-free survival for patients undergoing robot-assisted and laparoscopic radical prostatectomy over the follow-up durations.

Within the matched cohort, the median follow-up duration after RARP and LRP was 29 and 23 months, respectively. As shown in Table 3, no significant differences were detected between the two groups in terms of the median total serum PSA at postoperative 6 months and the last follow-up (*p* = 0.446 and *p* = 0.618, respectively). BCR appeared in 8 and 11 patients following RARP and LRP, respectively, over the periods covered by the follow-ups. The likelihoods of BCR-free survival of intermediate- and high-risk patients following RARP and LRP were also statistically similar after matching (*p* = 0.228, Figure 1).

**Figure 2:**
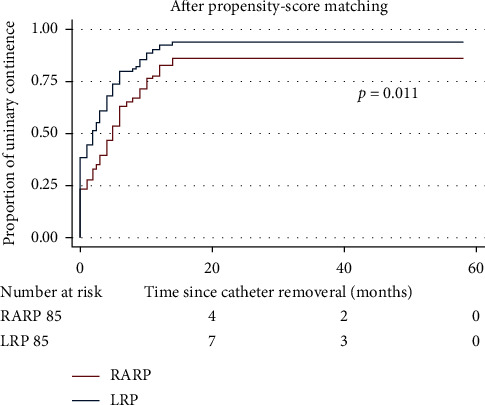
Kaplan–Meier curves showing the proportion of urinary continence (UC) in patients undergoing robot-assisted and laparoscopic radical prostatectomy over the follow-up durations. UC was defined as requiring no pad or preventively using one dry pad per day.

Foley catheter was routinely removed at postoperative 2 weeks regardless of the surgical approach. As described in Table 3, the proportions of patients reporting UC recovery at the moment of catheter removal (38.8% versus 23.5%, *p* = 0.031), postoperative 6 months (77.6% versus 63.5%, *p* = 0.043), and the last follow-up (94.1% versus 84.7%, *p* = 0.046) in the RARP group were significantly higher than those in the LRP group. Intriguingly, the difference in postoperative UC recovery obtained following RARP and LRP was gradually alleviated and close to be out of statistical significance. The patients in the RARP group achieved a significantly higher cumulative proportion of postoperative return to UC than those in the LRP group (*p* = 0.011, Figure 2).

As presented in Table 3, within the matched cohort, significant differences were also revealed between the RARP and LRP groups with respect to the median IIEF-5 score at postoperative 6 months and the last follow-up (*p* = 0.013 and *p* = 0.009, respectively), thus exhibiting the superiority of RARP over LRP in terms of erectile functional protection for men with intermediate- and high-risk PCa.

## 4. Discussion

Given its limited overall survival benefits and considerable adverse events, the role of RP in managing D'Amico low-risk PCa remains highly contentious [4, 6]. Meanwhile, the RP for D'Amico intermediate- and high-risk PCa could achieve favorable survival benefits from the further prevention of metastatic seeding of potentially lethal clones of PCa cells [11]. In 2020, RP was widely perceived as appropriate for men with intermediate- and high-risk PCa rather than those bearing D'Amico low-risk PCa [11]. Although RARP has been widely diffused for the surgical handling of localized PCa, the paucity of high-level evidence still triggers the controversy on the effects of RARP and LRP on the oncological and functional outcomes obtained after surgery. In addition, to date, no study has been immersed in comparing RARP and LRP for intermediate- and high-risk PCa. Meanwhile, the cogent evidence concerning the functional and oncological efficacy of RARP and LRP for intermediate- and high-risk localized PCa is of great clinical importance. The results of the present study revealed the superiority of RARP in functional preservation coupled with less postoperative ≤ Grade II complications than LRP without compromising cancer control for the management of intermediate- and high-risk PCa.

As regards the extended mean OT in the LRP group, the significant difference may be attributable to the fact that the robotic platform facilitates suturing, one of the most challenging procedures during the standard laparoscopic approach [27]; this advantage became more evident when comparing RARP and LRP for the intermediate- and high-risk patients enrolled in the present analysis. The similarities in the mean EBL and transfusion rates following RARP and LRP could be explained by the counterbalance between the contributing factors, including enhanced visualization, improved dexterity, and higher precision, to minimize bleeding during RARP and unfavorable factors leading to EBL, including more ePLNDs and nerve sparing procedures conducted in the RARP group. Johnson et al. [15] and Papachristos et al. [28] also achieved similar outcomes regarding EBL and OT after RARP and LRP in spite of the drastic variation in mean EBL and OT offered from different medical centers. The variation could be easily interpreted when taking the surgeons' experience and the patients and tumors' characteristics into account.

Although LRP and RARP are minimally invasive, the improved visualization and increased precision offered by the robotic platform could further reduce the operative invasiveness and the hazard of organ injuries [15, 27], which may translate into a significantly decreased proportion of overall and ≤ Grade II postoperative complications after RARP in the present analysis. However, the advantage mentioned above may be restricted by the high ePLND rate, which was associated with the occurrences of symptomatic lymphocele, which is the most frequent > Grade II complication in this study, in the RARP group, thus resulting in similar rates of > Grade II postoperative complications. The comparability of the incidence rates of postoperative > Grade II complications between the two groups in this analysis was consistent with that reported in a contemporary series [15, 18, 27–30] comparing RARP and LRP, thus demonstrating the similar operative safety of RARP and LRP for intermediate- and high-risk PCa in experienced hands.

The surgical approach to RP should be tempered with the critical significance of cancer control, especially when managing intermediate- and high-risk PCa. Consistent with the results reported in published analyses [17, 18, 31], no significant difference in the PSM rate was discovered in the comparison between RARP (17.6%) and LRP (24.7%) for intermediate- and high-risk PCa in the present study. The evaluated BMI and large prostate volume were considered as independent predictors of PSMs in men with organ-confined PCa [32], and cumulative evidence revealed that the margin status following RP is related to surgical experience [32, 33]. Fortunately, all these influencing elements were under stringent control with the application of the PM method in this single-center analysis, thus greatly contributing to the similarity in PSM rates after RARP and LRP. Of note, compared with the 15% mean rate of PSMs in RARP series involving more than 100 cases [32], 17.6% rate of PSMs acquired after RARP in the present analysis was relatively high, even in highly experienced hands, when removing intermediate- and high-risk PCa, thereby indicating that the more extensive the cancer is, the higher the possibility of positive margins is [32]. Although PSMs in RP specimens were in consistent correction with the enhanced risk of PSA relapse [34, 35], the long-term effects of PSMs on more robust clinical endpoints of the disease were variable and mostly depended on other variables, such as Gleason score, pathologic stage, and preoperative PSA [21, 36]. Intriguingly, most of these decisive factors, including Gleason score and preoperative PSA, were the basis of D'Amico's risk classifications. That is, the clinical endpoints, such as clinical recurrence rates, largely relied on preoperative baseline characteristics rather than PSMs after RP. In alignment with other series [18, 31] comparing RARP and LRP, the similarity in BCR-free survival obtained after RARP and LRP still existed, corroborating the equivalent potency of the two procedures in cancer control when managing intermediate- and high-risk PCa.

RP aims to completely eradicate localized PCa and, whenever possible, preserve UC and erectile function, that is, a trifecta outcome [6]. Urinary incontinence after RP is one of the most adverse events that negatively affects the quality of life of patients [27, 37]. Multiple pathophysiologic mechanisms contribute to the emergence of postprostatectomy incontinence (PPI). In addition to the biological/preoperative parameters encompassing patient age at the time of surgery, preexisting lower urinary tract symptoms, high BMI, abnormal bladder function, impairments of the integrity of anatomic supporting structures, and neural components during the RP procedure are crucial contributing factors to the development of PPI [37–40]. In the present study, preoperative/biological parameters were comparable between the RARP and LRP groups with the PM method being applied, but the robotic platforms allowed the enhanced preservation of membranous urethra and nerve branches and the reconstruction of the bladder neck, thus supporting the higher UC probability after RARP over the whole follow-up period. Many studies found that postoperative adjuvant radiotherapy adversely affects UC recovery following RP [41, 42], which may be attributed to a biological hypothesis that irradiation could lead to further damage and secondary inflammatory response to the surgical site. However, the impact of this factor affecting UC recovery in our analysis was extremely limited due to the statistically similar proportion of patients receiving adjuvant radiotherapy. In the prospective randomized controlled study reported by Porpiglia et al. [31], the UC rate after RARP was also significantly higher than that after LRP for localized PCa over a 5-year follow-up period. The results of the present study also corroborated the outcomes obtained in the first multicenter, randomized, patient-blinded controlled trial (LAP-01) [18], which demonstrated the improved postoperative return to UC of RARP over LRP. The advantage of robotic platforms with improved surgical vision and increased precision for preserving neurovascular structures could be greatly responsible for the superior erectile function recovery after RARP compared with that obtained after LRP.

Notably, several limitations should be taken into consideration when interpreting the conclusions. Structural shortages in data collection were inevitable in the retrospective setting of this analysis. The study population, although well-balanced between the two groups, was relatively small. The long-term oncological survival and functional recoveries could not be further evaluated over the relatively limited follow-up lengths. Certain complications may have also been undervalued, especially ≤ Grade II complications, in spite of the elaborative investigation of medical records and telephone interviews.

Despite these limitations, this study is the first one designed to assess the perioperative, functional, and oncological outcomes acquired after RARP and LRP for localized intermediate- and high-risk PCa thus far. The conclusions were drawn and strengthened on the basis of the comparability of all perioperative elements between the two arms and rigorous methodology.

## 5. Conclusions

For the surgical management of intermediate- and high-risk localized PCa, RARP tended to a lower risk of ≤ Grade II complications and superior functional preservation without cancer control being compromised than LRP. This conclusion needs to be further confirmed on the basis of prospectively randomized trials with large sample sizes and sufficiently long follow-ups.

## Figures and Tables

**Table 1:** Preoperative characteristics by surgery type before and after propensity score matching.

Variable	Before propensity score matching			After propensity score matching		
	RARP (*n* = 137)	LRP (*n* = 95)	*p* value	RARP (*n* = 85)	LRP (*n* = 85)	*p* value
Age, years, mean (SD)	65.4 (7.3)	68.0 (7.3)	0.010	65.5 (7.3)	67.2 (7.2)	0.138
BMI, kg/m^2^, mean (SD)	23.2 (3.5)	22.2 (3.8)	0.044	23.1 (3.6)	22.7 (3.8)	0.482
Diabetes mellitus (yes), *n* (%)	17 (13.4%)	15 (15.8%)	0.614	12 (14.1%)	14 (16.5%)	0.670
Hypertension (yes), *n* (%)	33 (26.0%)	23 (24.2%)	0.763	22 (25.9%)	20 (23.5%)	0.722
ASA score (≥3), *n* (%)	7 (5.5%)	7 (7.4%)	0.573	4 (4.7%)	5 (5.9%)	1.000
Preoperative total PSA, ng/mL, median (IQR)	17.5 (10.2, 34.1)	19.6 (10.0, 36.3)	0.495	18.4 (10.1, 34.0)	19.5 (10.2.35.5)	0.734
Prostate volume, mL, mean (SD)	38.4 (10.6)	43.9 (13.0)	0.001	39.7 (10.8)	42.8 (13.0)	0.093
Preoperative IIEF-5 score, median (IQR)	17 (14, 19)	15 (13, 18)	0.011	18 (14.3, 19)	16 (13, 18.75)	0.113
cTNM stage, *n* (%)			0.365			0.385
T1-T2a	52 (41.0%)	48 (50.5%)		37 (43.5%)	46 (54.1%)	
T2b	46 (36.2%)	29 (30.5%)		31 (36.5%)	25 (29.4%)	
T2c	29 (22.8%)	18 (19.0%)		17 (20.0%)	14 (16.5%)	
Biopsy Gleason score, median (IQR)	6 (5, 8)	7 (6, 8)	0.020	7 (5.75, 8)	7 (6, 8)	0.214
High risk^┿^, *n* (%)	62 (45.3%)	53 (55.8%)	0.115	43 (50.6%)	46 (54.1%)	0.645

SD: standard deviation; BMI: body mass index; ASA: American Society of Anesthesiologists; IIEF: International Index of Erectile Function; IQR: interquartile range. ^┿^According to the D'Amico risk classifications.

**Table 2:** Perioperative outcomes for RARP and LRP after propensity score matching.

Variable	RARP (*n* = 85)	LRP (*n* = 85)	*p* value
Operative time, min, mean (SD)	146.0 (40.4)	167.9 (34.2)	<0.001
Estimated blood loss, mL, mean (SD)	152.6 (60.5)	166.4 (78.2)	0.200
ePLND, *n* (%)	55 (64.7%)	50 (58.8%)	0.430
Nerve sparing procedures, *n* (%)	41 (48.2%)	28 (32.9%)	0.042
Open conversion, *n* (%)	0 (0%)	0 (0%)	-
Transfusion, *n* (%)	3 (3.5%)	7 (8.2%)	0.192
Postoperative pathology			
Pathological *T* stage, *n* (%)			0.345
pT2	49 (57.6%)	55 (64.7%)	
pT3	36 (42.4%)	30 (35.3%)	
Specimen Gleason score, median (IQR)	7 (5.5, 8)	7 (6, 8)	0.179
Positive surgical margin, *n* (%)	15 (17.6%)	21 (24.7%)	0.260
Number of removed lymph nodes, median (IQR)	10 (0, 16)	9 (0, 16)	0.523
Positive lymph nodes, *n* (%)	13 (15.3%)	10 (11.8%)	0.501
Postoperative complications, *n* (%)	8 (9.4%)	18 (21.2%)	0.033
≤ Grade II complications	6 (7.1%)	15 (17.6%)	0.036
> Grade II complications	2 (2.4%)	3 (3.5%)	1.000
Adjuvant radiotherapy, *n* (%)	37 (43.5%)	32 (37.6%)	0.435
Hospital stay, days, median (IQR)	14 (14, 15)	15 (14, 15)	0.563

ePLND: extended pelvic lymph nodes dissection; SD: standard deviation; IQR: interquartile range.

**Table 3:** Postoperative outcomes for RARP and LRP after propensity score matching.

Variable	RARP (*n* = 85)	LRP (*n* = 85)	*p* value
*Oncology*: *postoperative total PSA*, *ng/mL*			
Postoperative 6 months, median (IQR)	0.034 (0.020, 0.068)	0.025 (0.007, 0.060)	0.446
Last follow-up, median (IQR)	0.019 (0.009, 0.039)	0.022 (0.013, 0.053)	0.618

*Urinary continence*			
Continence on removal of catheter, *n* (%)	33 (38.8%)	20 (23.5%)	0.031
Continence at 6 months, *n* (%)	66 (77.6%)	54 (63.5%)	0.043
Continence at last follow-up, *n* (%)	80 (94.1%)	72 (84.7%)	0.046

*Erectile function*			
IIEF-5 score at postoperative 6 month, median (IQR)	14 (12, 16)	12 (11, 15)	0.013
IIEF-5 score at last follow-up, median (IQR)	14 (11, 16)	12 (11, 14)	0.009

PSA: prostate-specific antigen; SD: standard deviation; IIEF: International Index of Erectile Function; IQR: interquartile range.

## Data Availability

The datasets generated for this study are available upon request to the corresponding author.

## References

[1] Bray F., Ferlay J., Soerjomataram I., Siegel R. L., Torre L. A., Jemal A. (2018). Global cancer statistics 2018: GLOBOCAN estimates of incidence and mortality worldwide for 36 cancers in 185 countries. *CA: A Cancer Journal for Clinicians*.

[2] Deng W., Zhang C., Jiang H. (2021). Transvesical versus posterior approach to retzius-sparing robot-assisted radical prostatectomy: a retrospective comparison with a 12-month follow-up. *Frontiers in oncology*.

[3] Ilic D., Djulbegovic M., Jung J. H. (2018). Prostate cancer screening with prostate-specific antigen (PSA) test: a systematic review and meta-analysis. *BMJ*.

[4] Moschini M., Carroll P. R., Eggener S. E. (2017). Low-risk prostate cancer: identification, management, and outcomes. *European Urology*.

[5] Deng W., Liu X., Liu W. (2021). Functional and oncological outcomes following robot-assisted and laparoscopic radical prostatectomy for localized prostate cancer with a large prostate volume: a retrospective analysis with minimum 2-year follow-ups. *Frontiers in oncology*.

[6] Mottet N., van den Bergh R. C. N., Briers E. (2021). EAU-EANM-ESTRO-ESUR-SIOG guidelines on prostate cancer-2020 update. Part 1: screening, diagnosis, and local treatment with curative intent. *European Urology*.

[7] Tienza A., Akin Y., Rassweiler J., Gözen A. S. (2018). A match-pair analysis of continence in intermediate and high-risk prostate cancer patients after robot-assisted radical prostatectomy: the role of urine loss ratio and predictive analysis. *Prostate international*.

[8] Bill-Axelson A., Holmberg L., Garmo H. (2014). Radical prostatectomy or watchful waiting in early prostate cancer. *New England Journal of Medicine*.

[9] Wilt T. J., Jones K. M., Barry M. J. (2017). Follow-up of prostatectomy versus observation for early prostate cancer. *New England Journal of Medicine*.

[10] Chang A. J., Autio K. A., Roach M., Scher H. I. (2014). High-risk prostate cancer-classification and therapy. *Nature Reviews Clinical Oncology*.

[11] Costello A. J. (2020). Considering the role of radical prostatectomy in 21st century prostate cancer care. *Nature Reviews Urology*.

[12] Okegawa T., Omura S., Samejima M. (2020). Laparoscopic radical prostatectomy versus robot-assisted radical prostatectomy: comparison of oncological outcomes at a single center. *Prostate international*.

[13] Coughlin G. D., Yaxley J. W., Chambers S. K. (2018). Robot-assisted laparoscopic prostatectomy versus open radical retropubic prostatectomy: 24-month outcomes from a randomised controlled study. *The Lancet Oncology*.

[14] Bansal D., Chaturvedi S., Maheshwari R., Kumar A. (2021). Role of laparoscopy in the era of robotic surgery in urology in developing countries. *Indian Journal of Urology: IJU: journal of the Urological Society of India*.

[15] Johnson I., Ottosson F., Diep L. M. (2018). Switching from laparoscopic radical prostatectomy to robot assisted laparoscopic prostatectomy: comparing oncological outcomes and complications. *Scandinavian journal of urology*.

[16] Nossiter J., Sujenthiran A., Charman S. C. (2018). Robot-assisted radical prostatectomy vs laparoscopic and open retropubic radical prostatectomy: functional outcomes 18 months after diagnosis from a national cohort study in England. *British Journal of Cancer*.

[17] Asimakopoulos A. D., Pereira Fraga C. T., Annino F., Pasqualetti P., Calado A. A., Mugnier C. (2011). Randomized comparison between laparoscopic and robot-assisted nerve-sparing radical prostatectomy. *The Journal of Sexual Medicine*.

[18] Ju S., Holze S., Neuhaus P. (2021). Robotic-assisted versus laparoscopic surgery: outcomes from the first multicentre, randomised, patient-blinded controlled trial in radical prostatectomy (LAP-01). *European Urology*.

[19] Porpiglia F., Morra I., Lucci Chiarissi M. (2013). Randomised controlled trial comparing laparoscopic and robot-assisted radical prostatectomy. *European Urology*.

[20] Deng W., Zhang C., Jiang H. (2021). Robot-assisted vs. Laparoscopic radical prostatectomy for immediate- and high-risk localized prostate cancer: a propensity-score matched analysis. *Research Square*.

[21] Umari P., Eden C., Cahill D., Rizzo M., Eden D., Sooriakumaran P. (2021). Retzius-sparing versus standard robot-assisted radical prostatectomy: a comparative prospective study of nearly 500 patients. *The Journal of Urology*.

[22] Touijer A. K., Guillonneau B. (2004). Laparoscopic radical prostatectomy. *Urologic Oncology*.

[23] Rosen R. C., Riley A., Wagner G., Osterloh I. H., Kirkpatrick J., Mishra A. (1997). The international index of erectile function (IIEF): a multidimensional scale for assessment of erectile dysfunction. *Urology*.

[24] Dindo D., Demartines N., Clavien P. A. (2004). Classification of surgical complications: a new proposal with evaluation in a cohort of 6336 patients and results of a survey. *Annals of Surgery*.

[25] Deng W., Jiang H., Liu X. (2021). Transvesical retzius-sparing versus standard robot-assisted radical prostatectomy: a retrospective propensity score-adjusted analysis. *Frontiers in oncology*.

[26] Jo J. K., Jeong S. J., Oh J. J. (2018). Effect of starting penile rehabilitation with sildenafil immediately after robot-assisted laparoscopic radical prostatectomy on erectile function recovery: a prospective randomized trial. *The Journal of Urology*.

[27] Carbonara U., Srinath M., Crocerossa F. (2021). Robot-assisted radical prostatectomy versus standard laparoscopic radical prostatectomy: an evidence-based analysis of comparative outcomes. *World Journal of Urology*.

[28] Papachristos A., Basto M., Te Marvelde L., Moon D. (2015). Laparoscopic versus robotic-assisted radical prostatectomy: an Australian single-surgeon series. *ANZ Journal of Surgery*.

[29] Hakimi A. A., Blitstein J., Feder M., Shapiro E., Ghavamian R. (2009). Direct comparison of surgical and functional outcomes of robotic-assisted versus pure laparoscopic radical prostatectomy: single-surgeon experience. *Urology*.

[30] Menon M., Shrivastava A., Tewari A. (2002). Laparoscopic and robot assisted radical prostatectomy: establishment of a structured program and preliminary analysis of outcomes. *The Journal of Urology*.

[31] Porpiglia F., Fiori C., Bertolo R. (2018). Five-year outcomes for a prospective randomised controlled trial comparing laparoscopic and robot-assisted radical prostatectomy. *European urology focus*.

[32] Yossepowitch O., Briganti A., Eastham J. A. (2014). Positive surgical margins after radical prostatectomy: a systematic review and contemporary update. *European Urology*.

[33] Bravi C. A., Tin A., Vertosick E. (2019). The impact of experience on the risk of surgical margins and biochemical recurrence after robot-assisted radical prostatectomy: a learning curve study. *The Journal of Urology*.

[34] Jo J. K., Hong S. K., Byun S. S., Zargar H., Autorino R., Lee S. E. (2017). Positive surgical margin in robot-assisted radical prostatectomy: correlation with pathology findings and risk of biochemical recurrence. *Minerva urologica e nefrologica = The Italian journal of urology and nephrology*.

[35] Zhang L., Wu B., Zha Z., Zhao H., Jiang Y., Yuan J. (2018). Positive surgical margin is associated with biochemical recurrence risk following radical prostatectomy: a meta-analysis from high-quality retrospective cohort studies. *World Journal of Surgical Oncology*.

[36] Checcucci E., Veccia A., Fiori C. (2020). Retzius-sparing robot-assisted radical prostatectomy vs the standard approach: a systematic review and analysis of comparative outcomes. *BJU International*.

[37] Asimakopoulos A. D., Topazio L., De Angelis M. (2019). Retzius-sparing versus standard robot-assisted radical prostatectomy: a prospective randomized comparison on immediate continence rates. *Surgical Endoscopy*.

[38] Heesakkers J., Farag F., Bauer R. M., Sandhu J., De Ridder D., Stenzl A. (2017). Pathophysiology and contributing factors in postprostatectomy incontinence: a review. *European Urology*.

[39] Morozov A., Barret E., Veneziano D. (2021). A systematic review of nerve-sparing surgery for high-risk prostate cancer. *Minerva urology and nephrology*.

[40] Checcucci E., Pecoraro A. (2021). The importance of anatomical reconstruction for continence recovery after robot assisted radical prostatectomy: a systematic review and pooled analysis from referral centers. *Minerva urology and nephrology*.

[41] Nyarangi-Dix J. N., Steimer J., Bruckner T. (2017). Post-prostatectomy radiotherapy adversely affects urinary continence irrespective of radiotherapy regime. *World Journal of Urology*.

[42] Zaffuto E., Gandaglia G., Fossati N. (2017). Early postoperative radiotherapy is associated with worse functional outcomes in patients with prostate cancer. *The Journal of Urology*.

